# The Association between Serum Copper and Bone Mineral Density among Adolescents Aged 12 to 19 in the United States

**DOI:** 10.3390/nu16030453

**Published:** 2024-02-04

**Authors:** Haobiao Liu, Miaoye Bao, Mian Liu, Feidan Deng, Xinyue Wen, Ping Wan, Xue Lin, Guoqiang Dong, Zhaoyang Li, Jing Han

**Affiliations:** 1 Department of Occupational and Environmental Health, School of Public Health, Health Science Center, Xi’an Jiaotong University, Xi’an 710061, China; houbiu@stu.xjtu.edu.cn (H.L.); miaoyebao@stu.xjtu.edu.cn (M.B.); dengfeidan2000@stu.xjtu.edu.cn (F.D.); wenxy1116@stu.xjtu.edu.cn (X.W.); wanpingneo2018@stu.xjtu.edu.cn (P.W.); 1997601@stu.xjtu.edu.cn (X.L.); 2 Department of Bioengineering, College of Life Sciences, Fujian Normal University, Fuzhou 350117, China; 108032021045@student.fjnu.edu.cn; 3 Institute of Industrial Hygiene of Ordance Industry, Xi’an 710065, China; 521safe@163.com; 4 Key Laboratory of Environment and Genes Related to Diseases, Xi’an Jiaotong University, Ministry of Education, Xi’an 710061, China; 5 Global Health Institute, Health Science Center, Xi’an Jiaotong University, Xi’an 712000, China; 6 Key Laboratory for Disease Prevention and Control and Health Promotion of Shaanxi Province, Xi’an 710061, China

**Keywords:** serum copper, bone mineral density, adolescents, cross-sectional study, NHANES

## Abstract

Bone mineral density (BMD) is a key parameter widely used in the assessment of bone health. Although many investigations have explored the relationship between trace elements and BMD, there are fewer studies focused on serum copper and BMD, especially for adolescents. Using data extracted from the National Health and Nutrition Examination Survey, we applied a multiple-linear regression and smooth curve fitting to assess the relationship between serum copper and BMD. A total of 910 participants were finally included in this study. After adjusting for relevant covariates, serum copper was negatively associated with lumbar spine BMD (β = −0.057, 95% CI: −0.109 to −0.005), trunk bone BMD (β = −0.068, 95% CI: −0.110 to −0.026), pelvis BMD (β = −0.085, 95% CI: −0.145 to −0.024), subtotal BMD (β = −0.072, 95% CI: −0.111 to −0.033), and total BMD (β = −0.051, 95% CI: −0.087 to −0.016) (*p* < 0.05). In quartile analysis, the highest level of serum copper was associated with decreased BMD when compared with those at the lowest quartile (*p* < 0.05). The stratified analysis revealed a significant interaction between age and the effects of serum copper on trunk bone BMD (*p* = 0.022) and pelvis BMD (*p* = 0.018). Meanwhile, the higher level of serum copper was negatively associated with BMD in males, and gender modified the relationship (*p* < 0.001). Future longitudinal studies will be necessary for a more definitive interpretation of our results.

## 1. Introduction

Bone mineral density (BMD) is a bone mass parameter that reflects the level of bone mineral and is a major determinant of bone strength [1,2]. Widely utilized in assessing bone health, BMD is a key factor in diagnosing osteoporosis, predicting fracture risk, and evaluating treatment outcomes [3,4]. Studies have shown that low BMD is associated with a variety of conditions, including benign paroxysmal positional vertigo, obesity, and lumbar disc herniation [5,6,7,8]. In 2019, there were 438 thousand deaths and 16 million disability-adjusted life years attributable to low BMD, resulting in a significant burden of disease [9]. From 1990 to 2019, the prevalence of low BMD declined globally but increased in high-income regions of North America [10]. Therefore, exploring the factors influencing low BMD is essential for maintaining human health.

Copper is an essential trace element that plays a key role in many physiological processes, such as cell death, immune system stimulation, and angiogenesis [11,12,13]. Growing evidence suggests that an imbalance of copper content in the human body has been associated with a variety of diseases, such as stroke, Alzheimer’s disease, and Menke disease [14,15,16]. Recent studies described an association between serum copper levels and BMD, yet the findings from current research are inconclusive. A study conducted among children under 3 years of age revealed a positive correlation between serum copper levels and BMD [17]. In contrast, Rył A et al. found that serum copper concentration was negatively associated with BMD in aging men [18]. In addition, another study did not observe any significant association between serum copper and low BMD among elderly males [19].

Adolescence is a critical period for the development of the skeletal system, and at the end of puberty, bone mass is close to 95% of the peak bone mass in adults [20]. This means that understanding how various aspects of body composition influence bone mineral accumulation during adolescence so that more bone is gained and higher peak bone mass is achieved is essential for promoting optimal bone health and reducing fracture risk later in life [21,22]. Thus, it is particularly important to explore the influencing factors of BMD reduction during puberty to maintain bone health. However, no studies have been found on the relationship between serum copper and BMD in adolescents.

Therefore, the main purpose of this study is to explore the relationship between serum copper and BMD in adolescents using data from the National Health and Nutrition Survey (NHANES) in order to provide preliminary scientific evidence for the prevention of future osteoporosis and early detection and prediction of decreased bone density during adolescence.

## 2. Materials and Methods

### 2.1. Data Sources and Study Population

**Figure 1:**
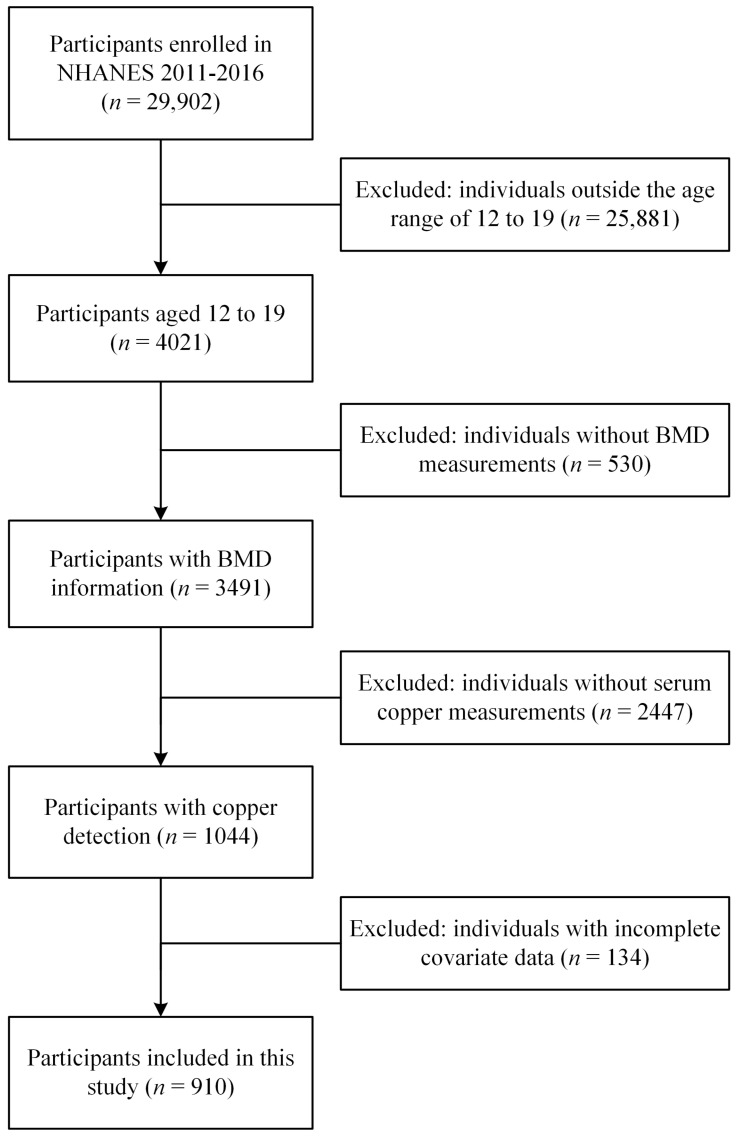
Flow diagram of the study participants included in NHANES 2011–2016.

The NHANES is a comprehensive program aimed at evaluating the health and nutritional status of both adults and children in the United States. The NHANES program began in the early 1960s and has been conducted as a series of surveys focusing on different population groups or health topics. This survey integrates both interviews and physical examinations. The interview section encompasses inquiries into demographic, socioeconomic, dietary, and health-related aspects. Meanwhile, the examination component entails a range of medical, dental, and physiological measurements, as well as laboratory tests conducted by proficient medical personnel. The data obtained from this program are instrumental in epidemiological and health sciences research, contributing to the development of robust public health policies, the direction and design of health programs and services, and the advancement of national health knowledge. In the current study, data from 29,902 participants included in the NHANES 2011–2016 database were extracted. Subsequently, data from individuals outside the age range of 12 to 19 (*n* = 25,881), those without BMD data (*n* = 530), those without serum copper data (*n* = 2447), and those with missing covariate data (*n* = 134) were excluded. Finally, a total of 910 participants were included in this study (Figure 1).

### 2.2. Measurement of Serum Copper

The serum copper level was measured using inductively coupled plasma dynamic reaction cell mass spectrometry (ICP-DRC-MS) [23]. In brief, serum samples were diluted at a ratio of 1:1:28 with water and a diluent containing gallium for multi-internal standardization. The liquid samples were then introduced into the ICP through a nebulizer and spray chamber, carried by a flowing argon stream. By coupling radio-frequency power into the flowing argon, plasma was generated, and the sample passed through a plasma region where thermal energy atomized and ionized the atoms. The resulting ions, along with the argon, entered the mass spectrometer. Subsequently, the ions traversed a focusing region, the dynamic reaction cell, and the quadrupole mass filter and were ultimately counted in rapid succession at the detector, enabling the determination of individual isotopes of an element. The lower limit of detection (LOD) for serum copper was 2.5 μg/dL. The measurement results below the LOD were placed by the LOD divided by the square root of 2.

### 2.3. Examination of BMD

Dual-energy X-ray absorptiometry (DXA) is widely recognized as the primary method for assessing body composition, owing to its rapidity, user-friendliness, and minimal radiation exposure. DXA scans yield measurements of bone and soft tissue across the entire body, including the arms, legs, trunk, and head. Additionally, bone measurements are obtained for specific areas such as the pelvis, ribs, thoracic spine, and lumbar spine. Trained and certified radiology technologists conducted the DXA examinations. Whole body scans were performed using the Hologic Discovery model A densitometers (Hologic, Inc., Bedford, MA, USA) equipped with Apex 3.2 software. Subsequently, all scans were analyzed using Hologic APEX version 4.0 software with the NHANES BCA option. The outcome variables in the present study were lumbar spine BMD, trunk bone BMD, pelvis BMD, subtotal BMD, and total BMD. Refer to the Body Composition Procedures Manual for a detailed laboratory procedure manual of the methods used [24].

### 2.4. Covariates

All covariates included and adjusted in this study were collected based on prior literature [25,26,27,28,29], including age, gender, race, poverty income ratio, body mass index (BMI), physical activity, total calcium, phosphorus, total protein, and total cholesterol. BMI was calculated as measured weight (kg) divided by the square of height (m^2^) and divided into underweight, normal, overweight, and obese according to the Centers for Disease Control and Prevention’s sex-specific 2000 BMI-for-age growth charts for the United States [30]. By multiplying the duration, frequency, and intensity of vigorous and moderate exercise during leisure time each week, the metabolic movement equivalent (MET, min/week) was calculated. Physical activity was then measured by MET and divided into low (<600 MET-min/week), medium (<1200 MET-min/week), and high (≥1200 MET-min/week). Further details on covariates can be found on the NHANES website [31].

### 2.5. Statistical Analysis

Continuous variables were expressed as means (standard deviation, SD), while categorical variables were expressed as numbers (percentages). All analyses in this study were performed using R software (version 4.2.2) and EmpowerStats (http://www.empowerstats.com, accessed on 16 January 2024), with a weighted NHANES sample according to the NHANES analysis guidelines [31]. Due to the skewed distribution, serum copper concentrations were natural logarithm (ln) transformed for further analyses. Meanwhile, serum copper concentrations were divided into four quartiles as follows: ≤4.51 μg/dL (Q1), 4.52–4.64 μg/dL (Q2), 4.65–4.78 μg/dL (Q3), and ≥4.79 μg/dL (Q4). We built the following two models to explore the association between serum copper and BMD. Model 1, adjusted for age, gender, and BMI. Model 2, adjusted for age, gender, BMI, race, poverty income ratio, physical activity, total calcium, phosphorus, total protein, and total cholesterol. Weighted multiple-linear regression models and smooth curve fittings were applied to assess the relationship between serum copper and BMD. Subgroup analyses were conducted according to age, gender, race, and BMI. We further performed a sensitivity analysis additionally adjusted for dietary and supplements of calcium, phosphorus, vitamin D, and protein. Statistical significance was set as *p* < 0.05.

## 3. Results

### 3.1. Characteristics of Participants

A comparison of sociodemographic and BMD characteristics of 910 participants between the overall and quartile groups at serum copper levels is shown in Table 1. The mean age was 15.44 ± 2.15, with 51.32% being male and 54.50% being non-Hispanic white.

Significant differences were observed in age, gender, race, BMI, phosphorus, total cholesterol, lumbar spine BMD, trunk bone BMD, subtotal BMD, and total BMD among the serum copper quartiles (*p* < 0.05). There were significant differences in age, gender, race, BMI, phosphorus, total cholesterol, lumbar spine BMD, trunk bone BMD, subtotal BMD, and total BMD in the serum copper quartiles (*p* < 0.05). Specifically, compared to the participants in the Q1 group, the Q4 group exhibited a higher proportion of females, as well as higher levels of BMI, total cholesterol, and lumbar spine BMD. On the contrary, when compared with participants in the Q1 group, those in the Q4 tend to be younger and exhibit lower levels of phosphorus, as well as reduced trunk bone BMD, subtotal bone BMD, and total BMD (*p* < 0.05, Table 1).

### 3.2. Associations between Serum Copper Level and BMD of Different Bony Sites

When age, gender, and BMI are adjusted in Model 1, per unit increment in ln-transformed copper was shown to be significantly associated with decreased levels of all five sites of BMD. After further adjusting for race, poverty income ratio, physical activity, total calcium, phosphorus, total protein, and total cholesterol in Model 2, per unit increment in ln-transformed copper was shown to be significantly associated with decreased levels of lumbar spine BMD (β = −0.057, 95% CI: −0.109 to −0.005), trunk bone BMD (β = −0.068, 95% CI: −0.110 to −0.026), pelvis BMD (β = −0.085, 95% CI: −0.145 to −0.024), subtotal BMD (β = −0.072, 95% CI: −0.111 to −0.033), and total BMD (β = −0.051, 95% CI: −0.087 to −0.016), respectively (Table 2).

**Figure 2:**
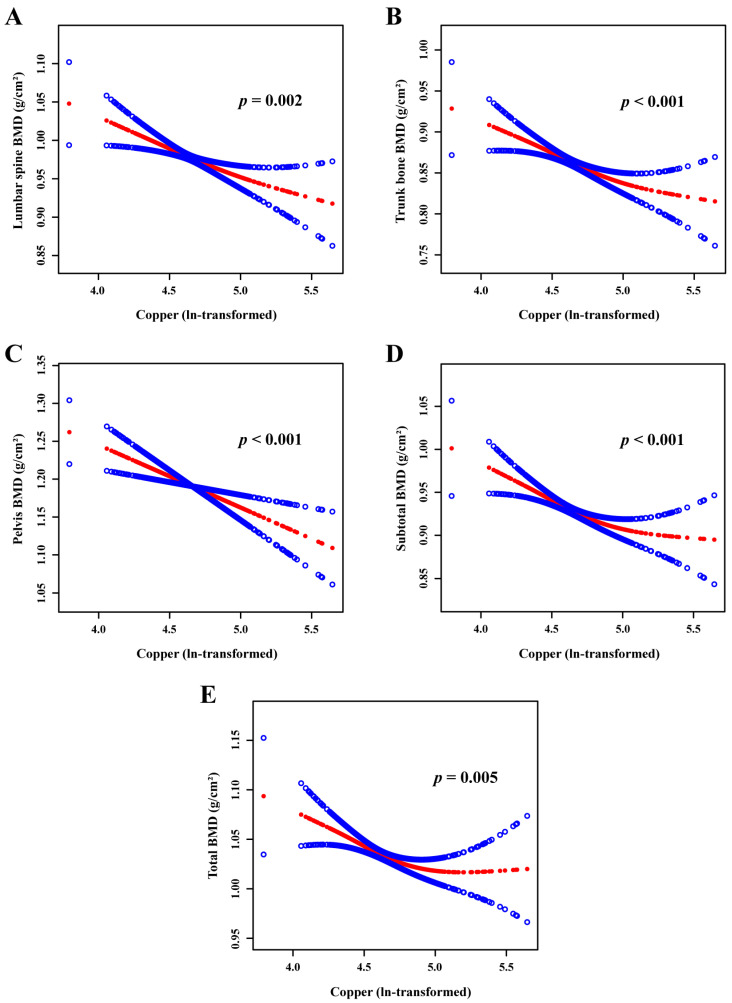
The relationship between serum copper concentration and lumbar spine BMD (**A**), trunk bone BMD (**B**), pelvis BMD (**C**), subtotal BMD (**D**), and total BMD (**E**). The red line represents the smooth curve fit between variables. The blue line represents the 95% confidence interval of the fit. Age, gender, BMI, race, poverty income ratio, physical activity, total calcium, phosphorus, total protein, and total cholesterol were adjusted. BMI, body mass index; BMD, bone mineral density.

Furthermore, smooth curve fitting (Figure 2) revealed a negatively linear relationship between the serum copper concentrations and lumbar spine BMD (*p* = 0.002), trunk bone BMD (*p* < 0.001), pelvis BMD (*p* < 0.001), subtotal BMD (*p* < 0.001), and total BMD (*p* = 0.005).

Additionally, negative correlations between serum copper levels and BMD indices were also observed when serum copper concentrations were converted to categorical variables. Compared with participants in the lowest quartile, those in the higher quartiles exhibited a significant association with lower BMD in the other four sites, except for lumbar spine BMD, when only adjusting for age, gender, and BMI (Model 1). After further adjusting for other relevant covariates (Model 2), the association continued to persist and become more pronounced at the other four sites as serum copper levels elevated. Notably, serum copper was also negatively associated with lumbar spine BMD but did not exhibit a stronger effect with higher copper levels (Table 2).

### 3.3. Subgroup Analysis of the Interactions and Sensitivity Analysis

Stratification analyses (Table 3) showed that in comparison to the 16–19 age group, the 12–15 age group exhibited a more pronounced negative correlation between serum copper levels and BMD. Meanwhile, there was a significant interaction between age and the effects of serum copper on trunk bone BMD (*p* = 0.022) and pelvis BMD (*p* = 0.018). The negative association between serum copper and lumbar spine BMD, trunk bone BMD, pelvis BMD, subtotal BMD, and total BMD was only observed among males. Meanwhile, significant interactions were also observed between gender and serum copper (*p* < 0.001). No interactions were found between race and serum copper or between BMI and serum copper in the five sites of BMD.

The sensitive analysis revealed that these negative correlations persisted, other than lumbar spine BMD, after additional adjustments for dietary and supplements of calcium, phosphorus, vitamin D, and protein (Table S1).

## 4. Discussion

Our study utilized data from the NHANES spanning from 2011 to 2016 to investigate the relationship between serum copper and BMD in adolescents aged 12 to 19 years. The results revealed a negative association between serum copper concentration and BMD.

Thus far, research on the relationship between serum copper and BMD has mostly focused on the adult population, while related epidemiological studies based on the population under 20 years old are very limited. For example, Qu, X. et al. reported that individuals with the lowest serum copper concentrations had lower BMD values, while those in the highest quartile of copper concentration had higher fracture rates, particularly among adult males, indicating adverse effects of excessive copper exposure on bone health [32]. However, Chaudhri et al. found a positive correlation between serum copper levels and BMD in postmenopausal women [33]. And one meta-analysis showed that Wilson’s disease, characterized by systemic copper overload, is directly associated with reduced bone mass, osteoporosis, and increased risk of fractures in both children and middle-aged individuals [34]. In contrast, one study by Eric D. Gaier et al. found that elderly males with low serum copper levels exhibited BMD similarly to the control group [35]. In addition, a study of children under 3 years of age unveiled a positive association between serum copper levels and BMD. In our study, we found that BMD decreased with higher serum copper levels among adolescents. Generally, the results of the existing studies are inconclusive, probably related to the age range of the population, as well as the mostly cross-sectional design, and further validation needs to be initiated in prospective studies in the future.

Currently, research on the effects of copper levels on bone metabolism is scarce, and the specific mechanisms by which copper influences BMD remain unclear. Copper serves as a cofactor for lysyl oxidase, an enzyme responsible for collagen fiber cross-linking and the maturation of the cross-linking process, the disruption of which leads to weakened bone structure [18]. Furthermore, recent evidence suggests that oxidative stress may be a contributing factor to bone damage [36]. However, high copper levels can lead to oxidative stress and the generation of free radicals, thereby contributing to chronic/degenerative diseases and carcinogenic effects, which are also detrimental [37]. Laboratory studies have indicated a U-shaped relationship between copper exposure and osteogenesis, with low-dose copper (0.1–1 µM) promoting osteogenesis and increasing bone nodule formation, while high-dose copper (50–100 µM) induces cytotoxic effects [38]. Therefore, suboptimal or excessive copper levels in the body may both pose risks for decreased bone mass. The association between high serum copper levels and lower BMD may be due to the fact that elevated copper levels lead to increased oxidative stress and free radical generation in the body, resulting in decreased BMD.

Furthermore, our study identified a gender-specific negative correlation between serum copper and BMD, which was only present in males. The observed phenomenon can be, at least in part, elucidated by disparities in sex hormone levels. Estrogen has direct effects on bone cells, osteoblasts, and osteoclasts. And estrogen can directly inhibit the activation of osteoclasts or block their activation through osteoblasts and T cells [39]. The ultimate effect of estrogen on the skeleton is to reduce bone remodeling and resorption while maintaining bone formation [39]. Notably, research has unveiled a significant correlation between serum estrogen levels and osteoporosis. Patients with osteoporosis exhibit markedly lower serum estrogen levels compared to their counterparts without the condition, further emphasizing the positive link between estrogen and BMD [40]. In addition, the SWAN Bone Cohort, a longitudinal study of 2335 women with an average age of 42–52 at baseline, demonstrated a significant acceleration of lumbar spine BMD loss in various ethnic groups during late menopause [41]. It is widely recognized that hormonal changes during adolescence play a critical role in bone growth and development [42]. In our study, females had higher serum copper levels than males, but there was no relationship between serum copper and BMD. Conversely, high copper levels in males were negatively correlated with BMD. This may be related to the higher estrogen levels in women aged 12–19 compared to men, which may mask the association between serum copper content and bone density. A future longitudinal study and in vivo research will be necessary for a more definitive interpretation of our results.

This study has several limitations. Firstly, due to the cross-sectional nature of the study, we cannot establish a causal relationship between serum copper levels and BMD, and therefore, further research is needed to confirm whether elevated serum copper levels directly affect BMD. Secondly, considering that this study was conducted in the US population, the current conclusions may not apply to other populations due to potential environmental, genetic, and racial differences. Finally, bone copper was not measured in NHANES, and therefore, we cannot determine the differences in bone copper levels between different groups.

## 5. Conclusions

In summary, our study provides preliminary clues for a negative association between serum copper and BMD in the adolescent population. Prospective studies are needed in the future to validate our research findings, and further exploration is needed to investigate the impact of gender differences on the association between serum copper and BMD.

## Figures and Tables

**Table 1:** Weighted characteristics of participants based on serum log-copper quartiles.

Characteristic	Serum Copper (μg/dL)					*p* Value
	Total	Q1 (≤90.6)	Q2 (90.7–103.2)	Q3 (103.3–119.3)	Q4 (≥119.4)	
Number of participants	910	227	215	228	240	
Age, years	15.44 (2.15)	15.83 (2.01)	15.52 (2.09)	14.84 (2.15)	15.57 (2.24)	0.007
Gender						<0.001
Male	456 (51.32)	166 (76.56)	109 (50.32)	101 (48.27)	80 (29.81)	
Female	454 (48.68)	61 (23.44)	106(49.68)	127 (51.73)	160 (70.19)	
Race						0.049
Non-Hispanic White	239 (54.50)	67 (56.24)	61 (58.97)	56 (53.69)	55 (49.10)	
Non-Hispanic Black	219 (13.58)	38 (8.99)	41 (9.97)	54 (13.08)	86 (22.29)	
Hispanic	297 (22.23)	79 (23.74)	71 (22.92)	78 (23.13)	69 (19.11)	
Other	155 (9.70)	43 (11.02)	42 (8.13)	40 (10.10)	30 (9.50)	
Poverty income ratio	2.39 (1.53)	2.39 (1.54)	2.56 (1.49)	2.34 (1.55)	2.29 (1.55)	0.438
BMI, Kg/m^2^						<0.001
Underweight	23 (2.94)	7 (3.69)	9 (5.96)	6 (1.57)	1 (0.56)	
Normal	512 (56.87)	161 (69.81)	141 (60.68)	125 (55.44)	85 (41.39)	
Overweight	171 (18.77)	34 (16.95)	40 (20.07)	43 (16.09)	54 (22.03)	
Obese	204 (21.42)	25 (9.54)	25 (13.28)	54 (26.89)	100 (36.02)	
Physical activity						0.336
Low	313 (30.29)	62 (23.90)	76 (31.61)	81 (29.77)	94 (35.97)	
Medium	125 (13.03)	34 (12.14)	30 (11.94)	32 (15.94)	29 (12.10)	
High	472 (56.68)	131 (63.95)	109 (56.44)	115 (54.29)	117 (51.93)	
Total calcium, mg/dL	9.61 (0.28)	9.61 (0.28)	9.63 (0.27)	9.65 (0.29)	9.55 (0.29)	0.063
Phosphorus, mg/dL	4.31 (0.65)	4.28 (0.66)	4.27 (0.64)	4.47 (0.64)	4.21 (0.63)	0.004
Total protein, g/dL	7.23 (0.40)	7.17 (0.37)	7.26 (0.42)	7.26 (0.41)	7.24 (0.39)	0.158
Total cholesterol, mg/dL	157.55 (31.38)	150.53 (29.00)	156.81 (34.15)	157.05 (28.51)	165.92 (31.87)	<0.001
Lumbar spine BMD, g/cm^2^	0.97 (0.15)	0.99 (0.15)	0.96 (0.15)	0.94 (0.16)	1.00 (0.16)	0.023
Trunk bone BMD, g/cm^2^	0.86 (0.12)	0.89 (0.12)	0.85 (0.13)	0.84 (0.12)	0.86 (0.12)	0.008
Pelvis BMD, g/cm^2^	1.19 (0.18)	1.20 (0.18)	1.18 (0.19)	1.17 (0.19)	1.21 (0.18)	0.209
Subtotal BMD, g/cm^2^	0.93 (0.12)	0.96 (0.12)	0.92 (0.12)	0.90 (0.11)	0.92 (0.10)	<0.001
Total BMD, g/cm^2^	1.03 (0.12)	1.06 (0.12)	1.02 (0.12)	1.01 (0.12)	1.04 (0.11)	0.006

Data are presented as mean (SD) or *n* (%). BMI, body mass index; BMD, bone mineral density.

**Table 2:** Association between serum copper and bone mineral density.

Variable	Model 1 β (95% CI)	Model 2 β (95% CI)
Lumbar spine BMD		
Continuous variable	−0.048 (−0.095, −0.002) *	−0.057 (−0.109, −0.005) *
Categorical variable		
Q1	Reference	Reference
Q2	−0.042 (−0.070, −0.013) **	−0.046 (−0.074, −0.017) **
Q3	−0.043 (−0.075, −0.010) *	−0.043 (−0.074, −0.012) **
Q4	−0.032 (−0.066, 0.001)	−0.043 (−0.079, −0.007) *
Trunk bone BMD		
Continuous variable	−0.057 (−0.095, −0.019) **	−0.068 (−0.110, −0.026) **
Categorical variable		
Q1	Reference	Reference
Q2	−0.026 (−0.048, −0.005) *	−0.030 (−0.053, −0.007) *
Q3	−0.035 (−0.062, −0.008) *	−0.036 (−0.060, −0.012) **
Q4	−0.041 (−0.067, −0.015) **	−0.052 (−0.079, −0.024) ***
Pelvis BMD		
Continuous variable	−0.067 (−0.121, −0.013) *	−0.085 (−0.145, −0.024) **
Categorical variable		
Q1	Reference	Reference
Q2	−0.023 (−0.057, 0.011)	−0.030 (−0.062, 0.003)
Q3	−0.033 (−0.070, 0.005)	−0.036 (−0.068, −0.004) *
Q4	−0.047 (−0.086, −0.007) *	−0.062 (−0.102, −0.022)**
Subtotal BMD		
Continuous variable	−0.063 (−0.100, −0.026) **	−0.072 (−0.111, −0.033) ***
Categorical variable		
Q1	Reference	Reference
Q2	−0.029 (−0.048, −0.009) **	−0.031 (−0.051, −0.010) **
Q3	−0.038 (−0.060, −0.015) **	−0.037 (−0.057, −0.016) **
Q4	−0.045 (−0.069, −0.021) ***	−0.053 (−0.077, −0.028) ***
Total BMD		
Continuous variable	−0.042 (−0.073, −0.011) **	−0.051 (−0.087, −0.016) **
Categorical variable		
Q1	Reference	Reference
Q2	−0.030 (−0.051, −0.010) **	−0.032 (−0.053, −0.011) **
Q3	−0.032 (−0.054, −0.009) **	−0.030 (−0.051, −0.009) **
Q4	−0.034 (−0.058, −0.011) **	−0.043 (−0.068, −0.018) **

Model 1, adjusted for age, gender, and BMI; Model 2, adjusted for age, gender, BMI, race, poverty income ratio, physical activity, total calcium, phosphorus, total protein, and total cholesterol. Serum copper concentrations were ln-transformed and included in the linear regression model. Results in bold indicate statistical significance. BMI, body mass index; CI, confidence interval; BMD, bone mineral density. Serum copper concentrations were ln-transformed and divided as follows: Q1, ≤4.51 μg/dL; Q2, 4.52–4.64 μg/dL; Q3, 4.65–4.78 μg/dL; Q4, ≥4.79 μg/dL. * *p* < 0.05; ** *p* < 0.01; *** *p* < 0.001.

**Table 3:** Stratified analyses of the association between serum copper concentrations and bone mineral density by age, gender, race, and BMI.

Characteristic	Lumbar Spine BMD		Trunk Bone BMD		Pelvis BMD		Subtotal BMD		Total BMD	
	β (95% CI)	*P*_interaction_	β (95% CI)	*P*_interaction_	β (95% CI)	*P*_interaction_	β (95% CI)	*P*_interaction_	β (95% CI)	*P*_interaction_
Age (years)		0.302		0.022		0.018		0.103		0.160
12–15 (*n* = 473)	−0.105 (−0.185, −0.025)		−0.124 (−0.182, −0.067)		−0.165 (−0.248, −0.082)		−0.115 (−0.175, −0.054)		−0.088 (−0.149, −0.028)	
16–19 (*n* = 437)	−0.055 (−0.119, 0.009)		−0.055 (−0.104, −0.007)		−0.060 (−0.126, 0.006)		−0.069 (−0.109, −0.029)		−0.045 (−0.085, −0.005)	
Gender		<0.001		<0.001		<0.001		<0.001		<0.001
Male (*n* = 456)	−0.177 (−0.245, −0.109)		−0.173 (−0.225, −0.122)		−0.211 (−0.283, −0.140)		−0.182 (−0.229, −0.134)		−0.161 (−0.204, −0.117)	
Female (*n* = 454)	0.005 (−0.049, 0.059)		−0.011 (−0.058, 0.035)		−0.015 (−0.088, 0.057)		−0.017 (−0.059, 0.025)		0.004 (−0.038, 0.046)	
Race		0.566		0.589		0.815		0.379		0.255
Non-Hispanic White (*n* = 239)	−0.065 (−0.142, 0.012)		−0.087 (−0.149, −0.025)		−0.103 (−0.189, −0.016)		−0.093 (−0.157, −0.030)		−0.065 (−0.122, −0.008)	
Non-Hispanic Black (*n* = 219)	−0.049 (−0.116, 0.017)		−0.063 (−0.120, −0.007)		−0.113 (−0.193, −0.033)		−0.052 (−0.097, −0.007)		−0.031 (−0.087, 0.026)	
Hispanic (*n* = 297)	−0.122 (−0.197, −0.046)		−0.106 (−0.168, −0.044)		−0.112 (−0.197, −0.027)		−0.111 (−0.159, −0.063)		−0.102 (−0.156, −0.049)	
Other (*n* = 155)	−0.086 (−0.191, 0.020)		−0.044 (−0.121, 0.034)		−0.053 (−0.143, 0.036)		−0.054 (−0.115, 0.006)		−0.016 (−0.089, 0.058)	
BMI		0.661		0.457		0.496		0.590		0.765
Underweight (*n* = 23)	−0.125 (−0.292, 0.043)		−0.036 (−0.197, 0.126)		0.090 (−0.208, 0.389)		−0.078 (−0.251, 0.095)		−0.089 (−0.264, 0.086)	
Normal (*n* = 512)	−0.058 (−0.116, −0.001)		−0.063 (−0.109, −0.017)		−0.086 (−0.151, −0.021)		−0.068 (−0.111, −0.025)		−0.048 (−0.090, −0.005)	
Overweight (*n* = 171)	−0.124 (−0.235, −0.014)		−0.132 (−0.216, −0.048)		−0.140 (−0.226, −0.054)		−0.112 (−0.195, −0.029)		−0.080 (−0.176, 0.015)	
Obese (*n* = 204)	−0.071 (−0.198, 0.057)		−0.100 (−0.207, 0.008)		−0.132 (−0.291, 0.026)		−0.117 (−0.213, −0.021)		−0.088 (−0.187, 0.010)	

BMD, bone mineral density. CI, confidence interval. BMI, body mass index.

## Data Availability

The data presented in this study are publicly accessible on the NHANES website [https://www.cdc.gov/nchs/nhanes/index.htm, accessed on 16 January 2024].

## Supplementary Materials

The following supporting information can be downloaded at https://www.mdpi.com/article/10.3390/nu16030453/s1. Table S1: Association between serum copper concentrations and bone mineral density after further adjustment of dietary and supplement of calcium, phosphorus, vitamin D, and protein (*n* = 885).
