# Supplementary material for: The Association between Serum Copper and Bone Mineral Density among Adolescents Aged 12 to 19 in the United States
Source: Nutrients. 2024 Feb 4;16(3):453. doi: 10.3390/nu16030453 (PMC10857197; doi:10.3390/nu16030453)
Supplement: Supplementary file 1 [file nutrients-16-00453-s001.zip › nutrients-2851166-supplementary.pdf]

Supplementary material

The Association between Serum Copper and Bone Mineral Density among Adolescents Aged 12 to 19 in the United States

Haobiao Liu [1,†], Miaoye Bao [1,†], Mian Liu [2,†], Feidan Deng [1], Xinyue Wen [1], Ping Wan [1], Xue Lin [1], Guoqiang Dong [3], Zhaoyang Li [1,4,*] and Jing Han [1,4,5,6,*]

[1] Department of Occupational and Environmental Health, School of Public Health, Health Science Center, Xi'an Jiaotong University, Xi'an 710061, China

[2] Department of Bioengineering, College of Life Sciences, Fujian Normal University, Fuzhou 350117, China

[3] Institute of Industrial Hygiene of Ordance Industry, Xi'an 710065, China

[4] Key Laboratory of Environment and Genes Related to Diseases, Xi'an Jiaotong University, Ministry of Education, Xi'an 710061, China

[5] Global Health Institute, Health Science Center, Xi'an Jiaotong University, Xi'an 712000, China

[6] Key Laboratory for Disease Prevention and Control and Health Promotion of Shaanxi Province, Xi'an 710061, China

* Correspondence

† These authors contributed equally to this manuscript.

Table S1. Association between serum copper concentrations and bone mineral density after additional adjustments for dietary and supplements of calcium, phosphorus, vitamin D, and protein (*n*=885).

Variable	β (95% CI)	*P* value
Lumbar spine BMD	-0.046 (-0.100, 0.008)	0.094
Trunk bone BMD	-0.056 (-0.100, -0.011)	0.016
Pelvis BMD	-0.065 (-0.128, -0.022)	0.043
Subtotal BMD	-0.062 (-0.102, -0.023)	0.004
Total BMD	-0.044 (-0.080, -0.008)	0.020

Serum copper concentrations were ln-transformed and included in the linear regression model. CI, confidence interval; BMD, bone mineral density.
